# The American and Colonial Journals: Surgery

**Published:** 1840-07

**Authors:** 


### SURGERY.

*Operation for Remedying an Anchylosis of the Hip-joint.* By Dr. J. Kearney.

"James Hall, an Irish labourer, aged 47 years, of healthy constitution, in October, 1829, suffered a severe injury by being caught between a vessel and the wharf. By this accident, the left thigh was fractured about its middle, and the right hip-joint severely contused. For the treatment of those injuries he was placed on his back, Boyer's apparatus applied to the left thigh, and the right thigh flexed, rotated outwards and abducted. The apparatus being badly adjusted, sloughing took place in the left groin, and all dressings were removed. No extension was kept up from this time, and the os femoris united two inches shorter than the right. The inflammation of the hip-joint proved very severe, and terminated in complete bony anchylosis.

"He was admitted under my care into the New York Hospital, November 10th, 1830. At this time, he could indeed walk, but with a painful effort, and the knees, in the act of progression, were separated two feet and a half. He was unable to support his family, and was desirous of having the deformity remedied. His general health was good. In consultation with my colleagues, Drs. Mott, Stevens, and Cheesman, I proposed to cut down on the os femoris, saw it off immediately above the less trochanter, and as this limb was two inches longer than the other, to remove as much as possible of the bone between the trochanter and the head, so as to make the two limbs, as nearly as I could, of the same length. This plan was assented to; and on the 24th of November, 1830, at 12 o'clock, the operation was performed in the following manner: An incision was made, six inches in length, in the course of the os femoris, beginning about an inch above the trochanter major. This was met about its middle by another from the front, three inches in length. The flaps were turned off, and the soft parts easily detached from the bone, so that in a short time, and with much less difficulty than I anticipated, my fingers were passed round the bone immediately above the trochanter minor. The division of the bone was attempted by the chain saw, but the instrument breaking, the section was completed with a saw recommended by Dr. Barton (North Am. Med. and Surg. Journ. for 1827, p. 292). This being accomplished, the limb was readily placed parallel with its fellow. Another section was made, and a wedge-shaped portion removed, the thickness of which at the outer part was about half an inch, and at the less trochanter three quarters of an inch. The removing a portion of bone of this shape I thought would enable me to keep the limb which had been greatly abducted more readily in situ. The wound was dressed with adhesive plaster and lint, and a bandage applied. The patient was now removed to his ward and placed on a firm mattress. The limb was kept in a proper position by a bandage to the feet.

"About the 1st of March the wound healed, and he was supported on crutches. He remained in the hospital until the beginning of May, 1831, when he left it of his own accord.

"*May*, 1833. My patient paid me a visit, walking well, and assisted only by a cane. He assured me that he could walk well enough if the left thigh gave him as little inconvenience as the one on which I had operated. But the left knee was somewhat stiff, as was the thigh, in consequence of the scars produced by the sloughs in the groin. He can rotate the right limb inward and outward; abduct it and flex it nearly to a right angle."

*Amer. Journ. of Med. Sciences.* No. 50. *Feb.* 1840.

---

*Case of Vesico-vaginal Fistula, successfully treated by an Operation.* By George Hayward, m.d., Surgeon to the Massachusetts General Hospital.

A married lady, ætat. 34, and of good health, consulted me on account of a vesico-vaginal fistula. Fifteen years ago, she was delivered, by means of instruments of her first child, which was dead, after having been in labour three days, during all of which time she passed no water. About ten days after her delivery an opening formed between the bladder and vagina, and since that period she has lost the retentive power of the bladder, and all the urine has escaped through the opening, except when a catheter has been introduced. Occasionally when in a horizontal posture there would be no escape of urine for two or three hours, though usually there was a continuous flow, but when in an erect position it was constantly dribbling, causing great inconvenience and distress. She had been eleven times pregnant since the accident, but had never gone her full period since the birth of her first child. It is not improbable that the fistula might have had some influence in the production of these repeated abortions. The only attempt that had been made to relieve her, consisted in the introduction of a catheter, which she wore for a considerable length of time, and touching the edges of the opening with caustic. Neither of these means afforded any relief; of late nothing had been done, and she regarded her case as almost hopeless. Upon examination, I found the fistula situated from an inch and a quarter to an inch and a third behind the urethra, a little on the left side. It was not large, barely sufficient to admit the end of my forefinger, and surrounded by a hardened edge, nearly of the consistence of cartilage. There was some degree of morbid sensibility in the lining membrane of the vagina, so that an examination was quite painful.

*Operation.* The patient was placed on the edge of a table, in the same position as in the operation for lithotomy. The parts being well dilated, I introduced a large bougie into the urethra and carried it back as far as the fistula. In this way I was able to bring the bladder downwards and forwards, so that the opening was brought fairly into view. The bougie being then taken by an assistant, I made a rapid incision with a scalpel around the fistula about a line from its edges, and then removed the whole circumference of the orifice. As soon as the bleeding, which was slight had ceased, I dissected up the membrane of the vagina from the bladder all around the opening, to the extent of about three lines. This was done partly with the view of increasing the chance of union, by presenting a larger surface, and partly to prevent the necessity of carrying the needles through the bladder. I then introduced a needle, about a third of an inch from the edge of the wound, through the membrane of the vagina and the cellular membrane beneath and brought it out at the opposite side at about an equal distance. Before the needle was drawn through, a second and a third were introduced in the same way; and there being found sufficient to close the orifice, they were carried through, and the threads tightly tied. Each thread was left about three inches in length. I should have remarked that I found no difficulty in introducing the needles by the hand, the fistulous opening having been brought so low down and so fairly in view. A short silver catheter constructed for the purpose was then introduced into the bladder, and the patient was conveyed to the bed and laid on her right side to prevent any urine from coming in contact with the wound. I found her in the evening, eight hours after the operation, quite comfortable. She had had some smarting for two or three hours, but this was soon gone; she complained a little of the catheter; all the water flowed through it and was received upon cloths. She was directed to live on thin arrow-root, milk and water, and a solution of gum arabic.

At the end of seventeen days from the operation I examined her again; the wound was entirely healed and apparently firm, and the soreness nearly gone. I advised her to introduce the catheter two or three times a day for some weeks; and on the following day she returned home by water, a distance of nearly two hundred miles.

*Amer. Journ. of Med. Sciences.* No. 48. *Feb.* 1840.

*Treatment of Ulcers in India.* By E. J. Downes, Esq.

My attention was first drawn to this subject whilst stationed at Allahabad in 1833, where I had numerous opportunities of witnessing cases of the deep and excavated ulcer, such as the bubo which has burrowed deeply, indolent ulcers of the legs, with ragged edges and irregular surfaces, &c.; and from the great difficulty frequently experienced in producing granulations, I was led to the adoption of a plan recommended by Mr. Stafford, which answered most completely, when various other remedies had failed. The treatment consists in pouring into the excavation melted wax of an adhesive quality, and just at that temperature when it is on the point of cooling (so as to avoid burning the patient), and this may be known by a portion of it clinging to the sides of the vessel, its beginning to thicken, and having somewhat of an opaque appearance. Mr. Stafford recommends four parts of white wax with one part of Venice turpentine, but I found a small quantity of spirits of turpentine answer every purpose. As soon as the wax has become solid in the ulcer, two or three strips of adhesive plaister, according to the extent of surface may be applied over it, so as to retain the wax in its situation; on the third day the dressing may be renewed. I was perfectly astonished at the rapidity with which healthy granulations were formed under these circumstances.

During my residence at Neemuch, in 1835, I was asked to prescribe for a valuable elephant belonging to Colonel Spiers; he told me that his elephant had a sore on her back, which had existed for eight months, rendering the animal perfectly useless, although, as the Colonel remarked, the Mahout tried all kinds of applications which he could think of to heal it up, as well as what he could get others of his tribe to prescribe for it, for a period of eight months. On examination I found an excoriated ulcer situated close behind the top of the animal's left shoulder extending backwards: the extent of the ulcer was five inches in length, two inches in breadth, to one and a half inch in depth, there was a sloughy appearance with a thin offensive discharge. I recommended a poultice for the first day, and recollecting the good results produced by the treatment I had previously adopted in similar cases of ulcers, I decided to try the wax in this instance. The dressing was removed after three days, and the alteration that had taken place in the appearance of the ulcer was really astonishing; fine healthy granulations were produced, and in the course of a month it completely healed.

*Indian Journal of Med. Science. July,* 1839.

---

*Removal of the Upper Jaw with a large portion of the Malar Bone.*
By Professor John Warren, of Boston.

The patient was a gentleman about sixty years of age, who for some time had been affected with fungus of the antrum, of a dreadfully painful kind, which must soon have proved fatal. The tumour was of a sugar-loaf form, occupying the right side of the face, and had forced its way through the cavities pertaining to the maxillary bone. The right eye was compressed and inflamed, and the cavities of the nostril partly filled by the tumour. Of course the support of the right eye and the right side of the right nostril and palate bones was removed, thus opening the nostril, mouth, and orbit into one common cavity. The patient supported this trying operation without a groan. He rose from the chair at the conclusion, and undressed himself before retiring to bed. The wound was closed by the twisted suture, and united by the first intention. In a fortnight he was well enough to leave the chamber and amuse himself with a spy-glass, using the organ which had been partly dissected from its socket in the operation he so lately passed through. The operation took place on the 17th of September, and on the 9th of October the delighted patient, thus almost miraculously saved from a horrible death, was able to leave the house.—*Boston Med. and Surg. Journal. Oct.* 16, 1839.

*Amer. Journ. of Med. Sciences.* No. 49. *Nov.* 1839.

